# COVID-19 associated cranial nerve neuropathy: A systematic review

**DOI:** 10.17305/bjbms.2021.6341

**Published:** 2021-08-11

**Authors:** Josef Finsterer, Fulvio Alexandre Scorza, Carla Alexandra Scorza, Ana Claudia Fiorini

**Affiliations:** 1 Clinic Landstrasse, Messerli Institute, Vienna, Austria; 2 Department of Neuroscience, Federal University of São Paulo/Paulista School of Medicine; 3 Department of Speech Therapy, Pontifical Catholic University of São Paulo, Paulista School of Medicine/Federal University of São Paulo, São Paulo, Brazil

**Keywords:** Cranial nerves, nerve conduction, neuropathy, SARS-CoV-2, COVID-19, Guillain Barre syndrome

## Abstract

The involvement of cranial nerves is being increasingly recognized in COVID-19. This review aims to summarize and discuss the recent advances concerning the clinical presentation, pathophysiology, diagnosis, treatment, and outcomes of SARS-CoV-2 associated cranial nerve mononeuropathies or polyneuropathies. Therefore, a systematic review of articles from PubMed and Google Scholar was conducted. Altogether 36 articles regarding SARS-CoV-2 associated neuropathy of cranial nerves describing 56 patients were retrieved as per the end of January 2021. Out of these 56 patients, cranial nerves were compromised without the involvement of peripheral nerves in 32 of the patients, while Guillain-Barre syndrome (GBS) with cranial nerve involvement was described in 24 patients. A single cranial nerve was involved either unilaterally or bilaterally in 36 patients, while in 19 patients multiple cranial nerves were involved. Bilateral involvement was more prevalent in the GBS group (n = 11) as compared to the cohort with isolated cranial nerve involvement (n = 5). Treatment of cranial nerve neuropathy included steroids (n = 18), intravenous immunoglobulins (IVIG) (n = 18), acyclovir/valacyclovir (n = 3), and plasma exchange (n = 1). The outcome was classified as “complete recovery” in 21 patients and as “partial recovery” in 30 patients. One patient had a lethal outcome. In conclusion, any cranial nerve can be involved in COVID-19, but cranial nerves VII, VI, and III are the most frequently affected. The involvement of cranial nerves in COVID-19 may or may not be associated with GBS. In patients with cranial nerve involvement, COVID-19 infections are usually mild. Isolated cranial nerve palsy without GBS usually responds favorably to steroids. Cranial nerve involvement with GBS benefits from IVIG.

## INTRODUCTION

Since the outbreak of the SARS-CoV-2 pandemic in December 2019 increasing evidence accumulated that not only the central nervous system (CNS) but also the peripheral nervous system (PNS) can be involved in this viral infection most frequently manifesting as lung disease (COVID-19) [1,2]. CNS involvement in COVID-19 includes viral meningitis, viral encephalitis, immune encephalitis, limbic encephalitis, acute, hemorrhagic, necrotizing encephalitis, acute, disseminated encephalomyelitis, transverse myelitis, multiple sclerosis, cerebral vasculitis, ischemic stroke, sinus venous thrombosis, cerebral vasoconstriction syndrome, intracerebral bleeding, or non-aneurysmatic subarachnoid bleeding. Manifestations of PNS involvement in the infection include neuropathy of cranial nerves, neuropathy of peripheral nerves, Guillain Barre syndrome (GBS) with all its subtypes (acute, inflammatory demyelinating polyneuropathy, acute, motor, axonal neuropathy, acute, motor and sensory, axonal ­neuropathy, Miller-Fisher syndrome, pharyngo-cervico-brachial variant, Bickerstaff encephalitis), myasthenia, myasthenic syndrome, myositis, and rhabdomyolysis [2,3]. Involvement of cranial nerves may occur as mono-neuropathy or polyneuritis cranialis, unilaterally or bilaterally, together with or without the involvement of peripheral nerves, and with or without CNS involvement. In the majority of cases with CNS/PNS involvement, cerebrospinal fluid (CSF) investigations for SARS-CoV-2 RNA are negative, suggesting that immunological reactions are the most common pathophysiological mechanism behind CNS/PNS involvement in COVID-19. Figures about the frequency of CNS/PNS involvement in COVID-19 are hardly available. This review aims to summarize and discuss previous and recent advances in the clinical presentation, pathophysiology, diagnosis, treatment, and outcome of SARS-CoV-2 associated neuropathies of cranial nerves.

## MATERIALS AND METHODS

A literature review in the databases PubMed and Google Scholar using the search terms “neuropathy,” “cranial nerves,” “optic nerve,” “olfactory nerve,” “oculomotor nerve,” “trochlear nerve,” “trigeminal nerve,” “abducens nerve,” “facial nerve,” “acoustic nerve,” “vestibulo-cochlear nerve,” “glossopharyngeal nerve,” “vagal nerve,” “accessory nerve,” “hypoglossal nerve,” and “nerves” together with “SARS-CoV-2,” “COVID-19,” and “coronavirus” was conducted. In addition, reference lists were checked for further articles meeting the search criteria. Included were articles which met the search criteria, reported original data (cases, case series), and were available as full articles. Excluded were articles available only as an abstract, proceedings, or review articles. Articles were also excluded because of limited data or absence of original data. Only articles in English were considered.

## RESULTS

**FIGURE 1:**
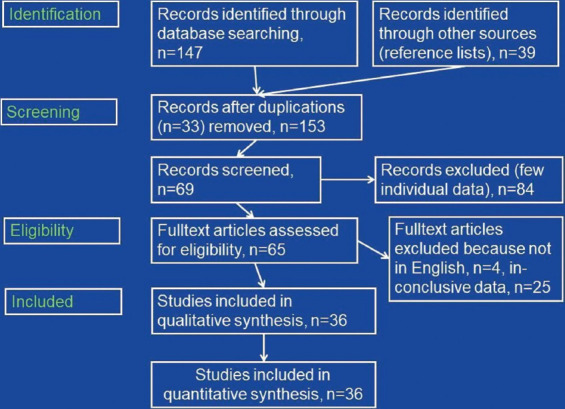
Flow chart detailing the search protocol and the results after application of inclusion and exclusion criteria.

Altogether 36 articles about SARS-CoV-2 associated neuropathy of cranial nerves describing 56 patients were retrieved as per the end of January 2021 (Figure 1) [4-39]. In 32 patients only cranial nerves without the involvement of peripheral nerves were affected (Table 1). In 24 patients GBS with involvement of cranial nerves were described (Table 1). Age, reported in 55 patients, ranged from 5 to 76 years (Table 1). Thirty-two patients were male and 23 were female (Table 1). In one patient gender was not reported (Table 1). There was female preponderance in the cohort with isolated cranial nerve involvement and vice versa male preponderance in the GBS cohort (Table 1). In 36 patients, a single cranial nerve was involved in 19 patients multiple cranial nerves were affected. In a single patient, the nerve involved was not specified (Table 1). In 15 patients one or more cranial nerves were bilaterally involved. Bilateral involvement was more prevalent in the GBS group as compared to the cohort with isolated cranial nerve involvement. Cerebral imaging was carried out in 38 patients and cranial nerve lesions were found in 20 of them (Table 1). Cerebral lesions were found in only two patients of whom one also had a cranial nerve lesion (Table 1). Cerebral imaging was normal in 17 patients (Table 1). Cranial nerve I was involved in three patients (Table 2), but several patients with anosmia were not included as the cause of anosmia remained elusive. Cranial nerve II was involved in seven patients, interestingly in none of the GBS group (Table 1). Cranial nerve III was compromised in 15 patients (Table 2). The trochlear nerve was involved in a single patient. The trigeminal nerve was compromised in 6 patients. Cranial nerve VI was involved in 17 patients (Table 2). The facial nerve was compromised in 29 patients. Cranial nerve VIII was damaged in two patients. Cranial nerves IX and X were injured in five patients each. Involvement of cranial nerve XI has not been reported. The hypoglossal nerve was compromised in 4 patients (Table 2). Treatment of cranial nerve neuropathy was reported in 52 cases and included steroids (n = 18), intravenous immunoglobulins (IVIG) (n = 18), acyclovir/valacyclovir (n = 3, two in combination with steroids), and plasma exchange (n = 1). Supportive measures were applied in four patients. Ten patients did not receive any treatment (Table 1). Therapy was not reported in four patients. The outcome was classified as “complete recovery” (complete resolution of cranial nerve impairment at the time of discharge or last follow-up) in 21 patients and as “partial recovery” (incomplete function of the compromised cranial nerve at the time of discharge or last follow-up) in 30 patients. Only a single patient died. In four patients, the outcome was not reported.

**TABLE 1:**
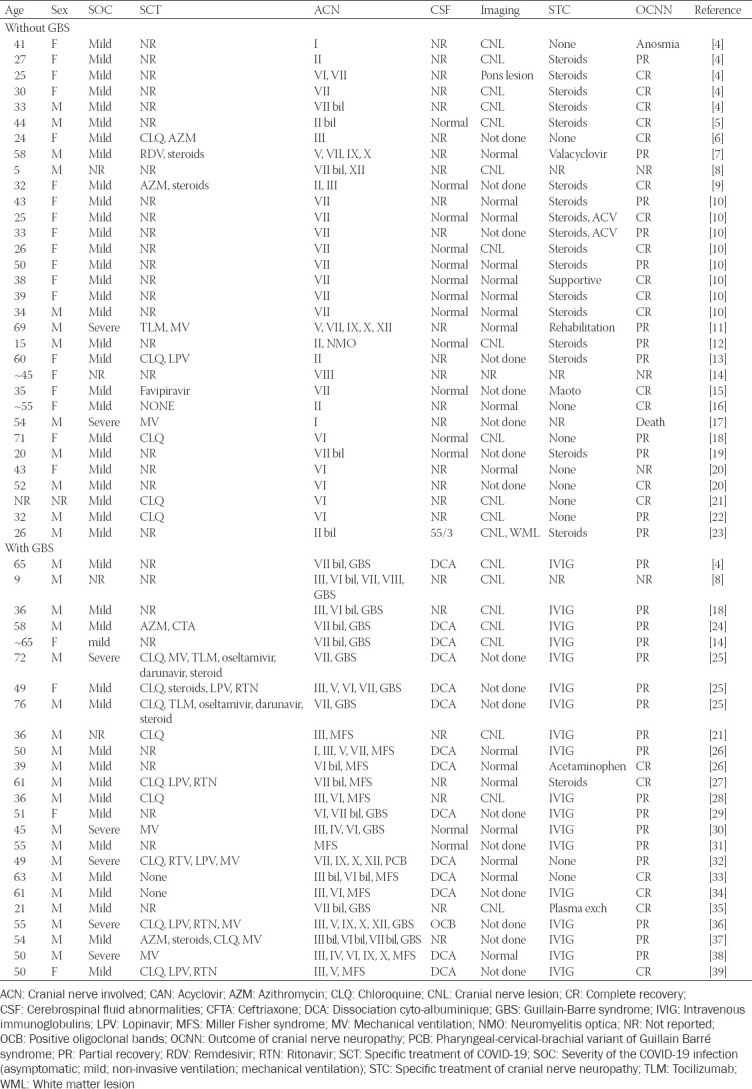
COVID-19 patients manifesting with involvement of a single or multiple cranial nerves

**TABLE 2:**
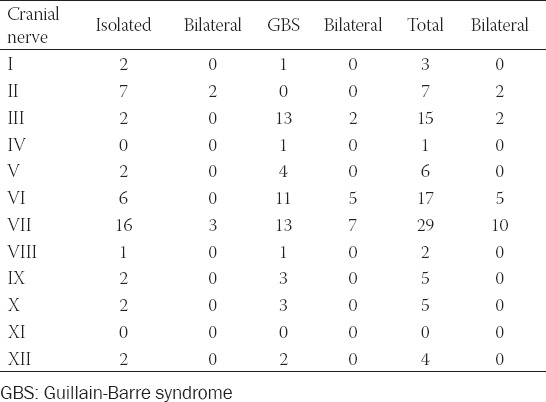
Number of COVID-19 patients with isolated cranial nerve involvement and GBS with cranial nerve involvement for each of the 12 cranial nerves

An illustrative case of cranial nerve involvement in COVID-19 was recently reported by Gogia et al. [7]. A 58-years-old male with mild COVID-19 developed left facial numbness, left facial dribbling, and mild “dysphagia” 4 days after onset of the viral infection [7]. Clinical exam revealed hypoesthesia in the distribution of the left trigeminal nerve and left facial palsy but excluded involvement of cranial nerves IX and X [7]. Cerebral MRI with contrast medium was non-informative. The patient refused CSF investigations. Facial and trigeminal nerve impairment partially resolved under valacyclovir during 7 days (Table 1) [7].

## DISCUSSION

This review shows that involvement of cranial nerves in the setting of COVID-19 infection is not rare and may be associated with GBS. Cranial nerves most frequently involved are cranial nerves VII, VI, and III manifesting as hypogeusia/ageusia, facial palsy, or ophthalmoparesis. COVID-19 in patients with associated cranial nerve involvement is usually mild but in patients with severe COVID-19 requiring mechanical ventilation, involvement of cranial nerves, and in particular GBS, may be missed. CSF investigations are usually normal in COVID-19 patients with isolated involvement of cranial nerves but show dissociation cyto-albuminique (DCA) or positive oligoclonal bands (OCB) in patients with GBS and concomitant cranial nerve involvement (Table 1). The majority of cases with isolated cranial nerve involvement benefit from steroids, whereas GBS cases with cranial nerve involvement benefit from IVIG. The outcome in isolated cases is usually fair with more patients reaching complete recovery than partial recovery. On the contrary, GBS patients with cranial nerve involvement more frequently achieve partial recovery as compared to complete recovery.

The pathophysiology of cranial nerve involvement remains elusive but it can be speculated that involvement of a cranial nerve results from the uptake of the virus into the intracellular space of neurons at a distal location followed by retrograde transport of the virus particles to the brain. An argument for this hypothesis is that in an autopsy study of 43 patients deceased from COVID-19 SARS-CoV-2 viral proteins were detected in cranial nerves originating from the lower brainstem and in isolated brainstem cells [40]. Furthermore, virus particles have been repeatedly found in neurons but also axons of cranial nerves in other autopsy studies [17]. Experimental studies indicate that SARS-CoV-2 indeed migrates retro-gradually within axons of cranial nerves to the CNS [41].

Whether anosmia/hyposmia and ageusia/hypogeusia are truly attributable to the involvement of cranial nerves I, VII, IX, and X, respectively, in each case is unknown, since only a small portion of these patients undergoes investigations of cranial nerve involvement and since it is conceivable that ageusia or anosmia results rather from the affection of appropriate receptors in mucous membranes than of the nerve. However, if the virus goes intercellularly, it is quite likely that the cranial nerves most frequently involved in COVID-19 are cranial nerves I, VII, IX, and X, as the prevalence of hyposmia/anosmia and hypogeusia/ageusia is high in several studies. Anyhow, as long as anosmia and hypogeusia are not confirmed by MRI or other means, they may not be classified as involvement of appropriate cranial nerves. Involvement of cranial nerves I should be diagnosed only if imaging demonstrates affection of the olfactory bulb or the fila olfactoria or if autopsy demonstrates the virus within olfactory neurons. If retrograde migration of the virus along olfactory or gustatory pathways can be confirmed, the frequency of cranial nerves I, VII, IX, and X needs to be re-assessed.

A second hypothesis explaining cranial nerve involvement relies on the assumption that immunological reactions against the virus secondarily affect neuronal structures due to epitope mimicry as in GBS. Arguments in favor of the radiculitis hypothesis are that cranial nerve involvement frequently occurs in patients with GBS and that patients with GBS and cranial nerve involvement may present with DCA or positive OCB on CSF investigations. An argument against the radiculitis hypothesis, however, is that imaging studies hardly revealed impairment of proximal portions of cranial nerves clinically involved in COVID-19. Interestingly, patients have been reported in whom MRI revealed enhancement of cranial nerves without corresponding clinical abnormalities [8]. Absence of cranial nerve II involvement in GBS patients could be explained by classification of the optic nerve as part of the CNS and not as cranial nerve. Whether involvement of the optic nerve in COVID-19 favours the development of demyelinating disease remains elusive but several cases with SARS-CoV-2 associated optic neuritis have been reported since the end of January 2021. Long-term evaluation of these patients is crucial to assess if SARS-CoV-2 triggers multiple sclerosis or neuromyelitis optica. As soon as a cranial nerve lesion becomes evident on a clinical exam in a patient with COVID-19 cerebral imaging is mandatory.

A third hypothesis explaining the involvement of cranial nerves in COVID-19 relies on the assumption that drugs given to treat COVID-19 could exhibit neurotoxic side effects particularly damaging cranial nerves. Drugs known to cause neuropathy and frequently given to COVID-19 patients include daptomycin [42], linezolid [43], lopinavir [44], ritonavir [45], hydro-chloroquine [46], cisatracurium [47], clindamycin [48], tocilizumab [49], and glucocorticoids [50]. An argument in favor of hypothesis three is that linezolid can cause optic and auditory nerve neuropathy [51, 52]. An argument against hypothesis three, however, is that COVID-19 in patients with cranial nerve involvement is usually mild and does not require aggressive treatment with any of the neurotoxic compounds. To which degree tocilizumab contributed to the development of cranial nerve palsies in the three patients who received it [11,25], remains speculative.

Treatment of cranial nerve involvement relies on the application of anti-COVID-19 drugs and more specifically on the application of steroids, particularly in patients with isolated cranial nerve involvement, of IVIG, particularly in patients with GBS and cranial nerve involvement, and of plasma exchange in single cases with GBS and cranial nerve involvement. In single cases virostatics, such as acyclovir or valacyclovir, were tried with limited effect. Recently, stimulation of cranial nerves was suggested to be used for therapeutic purposes of COVID-19 [53]. In patients with severe COVID-19 under mechanical ventilation, transcutaneous, non-invasive vagal nerve stimulation has been shown to improve lung functions [53].

Limitations of the review were that detailed individual data from several studies were not available. In a retrospective study of 50 COVID-19 patients, five patients with ophthalmoparesis and two patients with facial palsy were reported. Unfortunately, no detailed individual data were provided for these patients, why they were not included in the present evaluation [54]. In a retrospective study of 841 COVID-19 patients, optic neuritis was reported in a single patient [55]. For this particular patient, no individual data were provided. Lin et al. reported four patients with olfactory bulb abnormalities on MRI but did not present details required to be included in Table 1 [21]. The patient with anosmia and motor deficits reported by Aragão et al. (patient-2) [56] was not included as a work-up for GBS, respectively, neuropathy is missing and delineation between cranial nerve involvement plus GBS or only cranial nerve involvement is not feasible.

## CONCLUSION

This review shows that all cranial nerves can be involved in COVID-19 but most frequently cranial nerves VII, VI, and III. Involvement of cranial nerves in COVID-19 may go along with or without GBS. As per the end of January 2021, 32 patients with isolated cranial nerve involvement and 24 patients with GBS and involvement of cranial nerves were reported. Cerebral imaging in patients with cranial nerve involvement is crucial as it may demonstrate a lesion not only in clinically affected but also clinically unaffected cranial nerves. In patients with cranial nerve involvement COVID-19 infections are usually mild. Isolated cranial nerve palsy without GBS usually responds favorably to steroids. GBS with cranial nerve involvement benefits from IVIG.
